# Depth filter material process interaction in the harvest of mammalian cells

**DOI:** 10.1002/btpr.3329

**Published:** 2023-02-23

**Authors:** Maria Parau, James Pullen, Daniel G. Bracewell

**Affiliations:** ^1^ Department of Biochemical Engineering University College London London UK; ^2^ Research and Development FUJIFILM Diosynth Biotechnologies (FDB) Billingham UK

**Keywords:** confocal imaging, depth filter, harvest, mAbs, protein A chromatography

## Abstract

Upstream advances have led to increased mAb titers above 5 g/L in 14‐day fed‐batch cultures. This is accompanied by higher cell densities and process‐related impurities such as DNA and Host Cell Protein (HCP), which have caused challenges for downstream operations. Depth filtration remains a popular choice for harvesting CHO cell culture, and there is interest in utilizing these to remove process‐related impurities at the harvest stage. Operation of the harvest stage has also been shown to affect the performance of the Protein A chromatography step. In addition, manufacturers are looking to move away from natural materials such as cellulose and Diatomaceous Earth (DE) for better filter consistency and security of supply. Therefore, there is an increased need for further understanding and knowledge of depth filtration. This study investigates the effect of depth filter material and loading on the Protein A resin lifetime with an industrially relevant high cell density feed material (40 million cells/ml). It focuses on the retention of process‐related impurities such as DNA and HCP through breakthrough studies and a novel confocal microscopy method for imaging foulant in‐situ. An increase in loading of the primary‐synthetic filter by a third, led to earlier DNA breakthrough in the secondary filter, with DNA concentration at a throughput of 50 L/m^2^ being more than double. Confocal imaging of the depth filters showed that the foulant was pushed forward into the filter structure with higher loading. The additional two layers in the primary‐synthetic filter led to better pressure profiles in both primary and secondary filters but did not help to retain HCP or DNA. Reduced filtrate clarity, as measured by OD600, was 1.6 fold lower in the final filtrate where a synthetic filter train was used. This was also associated with precipitation in the Protein A column feed. Confocal imaging of resin after 100 cycles showed that DNA build‐up around the outside of the bead was associated with synthetic filter trains, leading to potential mass transfer problems.

## 1 INTRODUCTION

Advances in upstream development have led to high titres (5–10 g/L),^1^ which have put pressure on the protein A chromatography to process increased protein mass.^2^ In addition, the high cell density required to achieve high titres leads to increased impurity load experienced by the chromatography resin. Publications have shown that the choice of harvest can affect the HCP profile in Protein A eluate^3^ and that HCP that co‐elute with the mAb are more challenging to remove at high cell density.^4^ DNA has also been shown to be problematic for resin fouling due to chromatin‐histones complexes.^5^, ^6^ However the cell culture used had a viability of 20–50%, suggesting chromatin might only be problematic at low viability. The choice of upstream depth filter type has also been shown to cause peak broadening in Protein A elution, suggesting carry‐over of DNA.^7^

Resin lifetime is negatively affected by the CIP stage due to ligand degradation. However, fouling due to the feed material has been identified to have the most impact on resin lifetime.^8^, ^9^ Mechanisms contributing to resin lifetime are ligand leaching, ligand degradation and coating of the resin surface, and pore blocking.^8^ Studies have found that fouling due to pore blocking and reduced availability of binding sites was of most significant concern^9^; HCP had a higher affinity to the resin than the mAbs and that the typical CIP conditions could not remove all fouling impurities.^10^ Other studies have shown that culture fluid containing mAb products caused more fouling than null‐cell culture fluid.^11^ A study on AEX resin^12^ found that foulant forms a layer on the surface of the resin but does not significantly penetrate into the resin bead. However, it blocks the pore entrance and reduces the available surface area for diffusion, increasing resistance to mass transfer.

Therefore, there is interest in removing process‐related impurities such as DNA and HCP during the harvest stage to maintain long resin lifetimes. Depth filters have the potential to remove impurities at the harvest stage. In addition to removal of material based on size exclusion, depth filters have been shown to remove soluble impurities by adsorption through hydrophobic, ionic, and other interactions^13^ to remove endotoxin^14^ and DNA.^15^, ^16^ Furthermore, it has been shown that positively charged depth filters can reduce HCP and turbidity of Protein A chromatography eluate.^17^, ^18^ More recent publications have also described the ability of charged filter media to remove HCP and DNA.^18^, ^19^, ^20^, ^21^, ^22^ However, it is important to note that these studies have used either model protein solutions or cell density below 10 million cells/ml. Therefore while informative, they are not representative of an industrial relevant high cell density process.

In previous work,^23^ we have shown that there was an immediate breakthrough and no HCP removal at high cell density (~30 million cells/ml). Furthermore, DNA removal depended on input concentration, which was a factor of cell culture viability. We also showed through confocal imaging that the material of secondary depth filter affected the distribution of the foulant within the two layers on the filters and that the increased capacity of the secondary filters was related to higher intensity measurements in the confocal images. This study investigates the performance of three depth filtration trains where throughput is controlled. This study hypothesizes that the additional layers in the primary‐synthetic filter lead to higher capacity in the primary filter, hence protecting solids carry‐over to the secondary filter. In turn, the secondary filter can remove more process‐related impurities such as DNA and HCP. The impact of each harvest train on Protein A resin lifetime is also investigated.

## 2 MATERIALS AND METHODS

### 2.1 Cell culture conditions

A mAb feedstock produced in Chinese Hamster Ovary (CHO) cells was provided by FUJIFILM Diosynth Biotechnologies utilizing their Apollo X™ platform. The material was produced in shake flasks with a 2 L working volume, using a proprietary Fed‐batch process. Cells were seeded at a density of 0.5 million cells/ml, incubated at 37°C and harvested on day 14. The cell culture characteristics can be found in Table 1.

**TABLE 1:** Description of the three filtration trains and the characteristics of the CHO cell culture at harvest

					Cell culture characteristics		
Filter train	Filtration train	Filter operation & loading	Primary filter loading (L/m^2^)	Secondary filter loading (L/m^2^)	Total cell count (million cells/ml)	Viability (%)	IgG (mg/ml)
**A**	**270 cm** ^**2**^ **D0HC + 2x 23 cm** ^**2**^ **X0HC**	**Max pressure – 30 psi (Low load)**	68	101	46.3	75	6.0
**B**	**270 cm** ^**2**^ **D0SP + 2x 23 cm** ^**2**^ **X0SP**	**Same loading as A** **(Low load)**	66	99	44.8	67	6.1
**C**	**270 cm** ^**2**^ **D0SP + 2x 23 cm** ^**2**^ **X0SP**	**Increased loading (+30% than A)** **(High load)**	104	226	42.9	69	6.9

### 2.2 Depth filtration

Three depth filtration trains were tested, one using the Millistak+ HC series and two using the SP series, where filter loading was controlled. Details of each filtration train are in Table 1. Each train consisted of 1 x 270 cm^2^ primary filter (D0HC/SP) and 2 x 23 cm^2^ secondary (X0HC/SP) pods operated in parallel. The primary and secondary pods were operated individually, and an intermediate pool was collected. The post‐secondary depth filtrate was further clarified using a 0.2 μm SartoPore® 2 (Sartorius) with a surface area of 0.03 m^2^ and stored at ‐20°C in 40 ml aliquots.

### 2.3 Protein A resin lifetime studies

The aliquots from the depth filtration experiments above were used for the Protein A lifetime studies. For each condition, 100 cycles were performed. The studies were conducted using a 1 ml HiTrap column prepacked with MabSelect Sure LX and the AKTA Avant (Cytiva) chromatography system. Eluates were collected every cycle and neutralized with 200 μl 2 M Tris‐Base. Precipitation was observed in some loading material and was removed by centrifugation at 500 rpm for 5 min. All feeds were clarified using a 0.2 μm syringe filter before loading.

### 2.4 Protein A method

The column was washed with 6CV of 20 mM sodium phosphate, 150 mM NaCl pH 7.4. The column was loaded with the clarified harvest up to 45 g protein/L resin and then followed by two washes using 5CV 20 mM sodium phosphate, 500 mM NaCl pH 7.4, and then 3CV of 50 mM sodium phosphate pH 6. Next, the mAb was eluted with 6CV of 50 mM sodium acetate pH 3.5, and the peak was collected at 50‐20 mAU. The column was then stripped using 2CV of 100 mM Acetic acid, washed with 2CV of purified water, and then sanitized with 3CV 0.1 M NaOH and 15 min contact time (no flow) before being regenerated with the equilibration buffer. The column was stored in 20% ethanol when not in use. The linear flowrate used was 300 cm/h except for the load and the elution, which were done at 200 and 100 cm/h, respectively.

### 2.5 Confocal imaging of depth filters and resin

The method for staining and imaging the depth filters is described in full in a previous publication.^23^ The resin staining assay was adapted from.^24^ After 100 cycles were completed on the 1 ml HiTap columns, the top was cut, and the resin removed. A 20% (v/v) resin slurry was made using MilliQ Water. The Proteostat fluorescent dye was prepared according to the manufacturer's instructions, and 2 μl of the dye was added to 98 μl resin slurry and incubated for 20 min protected from light. Samples were prepared in triplicates. For the fluorescence intensity, samples were added to a black 96‐well plate, and measurements were determined at 550 and 600 nm excitation and emission, respectively. The same sample prep method was repeated with PicoGreen® fluorescent dye. The only difference was that fluorescence intensity was determined at 480 and 520 nm excitation and emission, respectively.

The samples were prepared in the same way for confocal imaging. However, a flow cell was used to house the resin samples. A Leica TCS SPE inverted CLSM (Leica Microsystems) was used to visualize particular areas of fluorescence in resin samples. Microscope settings were the same throughout all experiments: magnification 40× with oil immersion, gain 900. Resin samples stained with the Proteostat dye were imaged at excitation wavelength 532 nm, emission wavelength 600 nm, and laser intensity 10%. Resin samples stained with the PicoGreen® dye were imaged at excitation wavelength 488 nm, emission wavelength 500‐550 nm, and laser intensity 20%.

Image analysis of resin fluorescence distribution was performed in ImageJ software. Each resin bead in the image was measured individually, and the Mean gray value was measured, defined in ImageJ as the Average gray value within the selection. This is the sum of the gray values of all the pixels in the selection divided by the number of pixels reported in calibrated units. The normal distribution was calculated for each resin sample and dye.

### 2.6 DNA, HCP and mAb quantification

DNA concentration was measured using the QuantIT™ PicoGreen® dsDNA Kit (ThermoFisher Scientific), as per the manufacturer's protocol. Total protein concentration was measured using Pierce™ Rapid Gold BCA Protein Assay Kit (ThermoFisher Scientific), as per the manufacturer's protocol.

Optical density at 600 nm was measured using a sample volume of 200 μl in a UV transparent 96‐well microplate.

IgG quantification was performed by Protein A HPLC. A POROS A20 column (Thermo Scientific) was connected to an Agilent 1200 Series HPLC (Agilent, Santa Clara, CA.). The sample injection volume was 100 μl. Equilibration was performed with 20 mM sodium phosphate, 300 nM NaCl pH 7.2. Elution was performed with 20 mM sodium phosphate, 300 nM NaCl pH 2.5. The flow rate was 1.5 ml/min. Concentration was calculated using an in‐house IgG1 standard.

HCP concentration for the filtration samples was calculated as per the following equation: [HCP] = [Total Protein] ‐ [IgG].

## 3 RESULTS AND DISCUSSION

### 3.1 Pressure, filter type, and filter loading

Table 1 describes the variables in the three filtration trains and the cell culture characteristics. The two filtration stages were operated individually, with an intermediate pool collected between the primary and secondary stage. Filtration train A and B are made up of cellulose‐based and synthetic‐based depth filters, respectively. They have a similar filter loading which was based on the max pressure of 2 bar in the cellulose‐based filter. For filtration train C, also synthetic‐based, a loading increase of 1/3 was decided upon as to see significant differences in the filtrate quality and still be a relevant range for manufacturing. Only three filtration trains were investigated due to material shortages.

**FIGURE 1:**
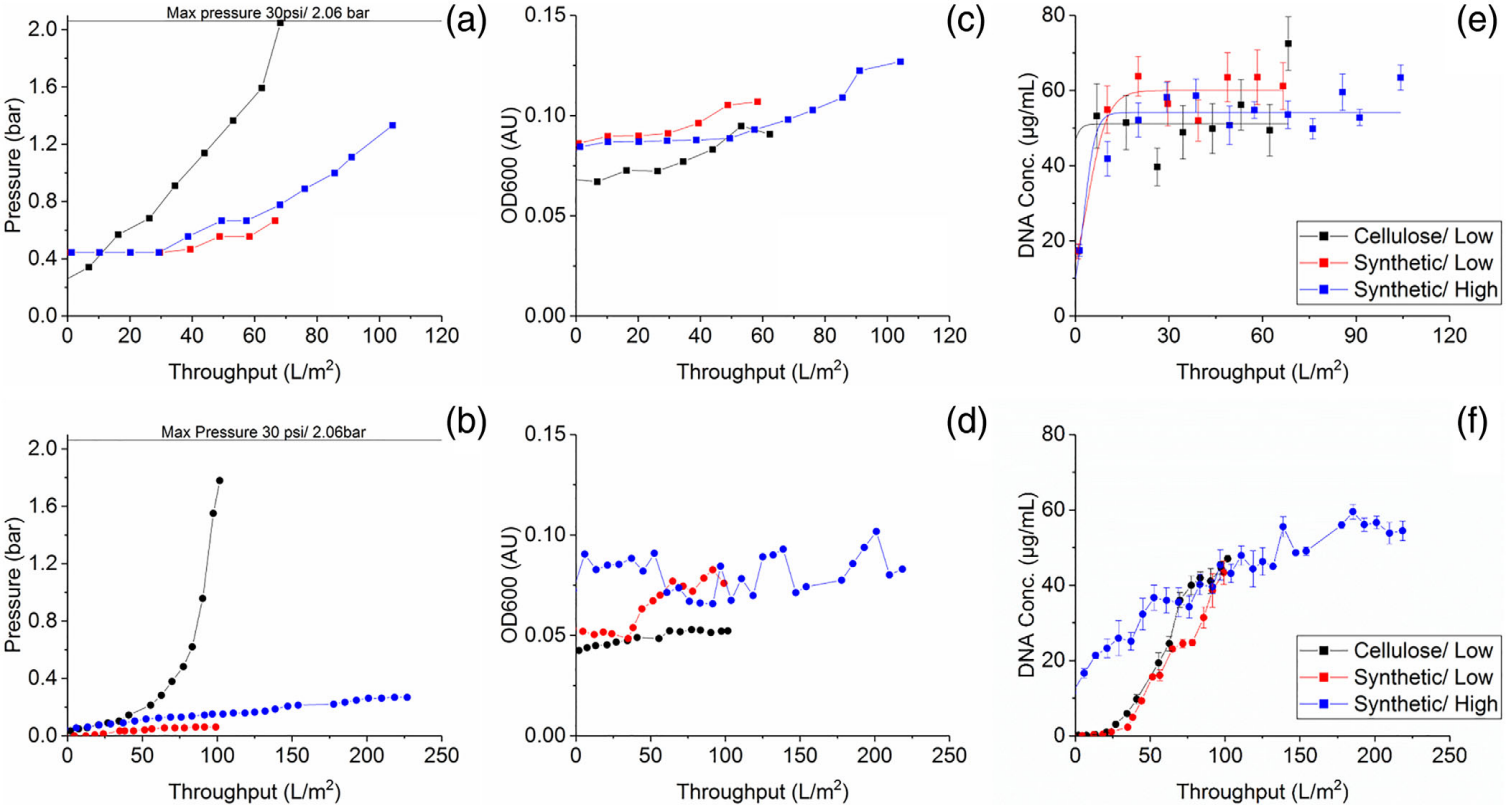
Pressure (a‐b), OD600 (c‐d) and DNA (e‐f) breakthrough after the primary (top) and secondary (bottom) depth filters. Details of filtration train and cell culture are in Tables 1 & 2. Error bars represent 1SD of 3 repeats of the PicoGreen assay. Pressure values in (a) are due to a different pressure gauge used. Lines of best fit in (b and e) are plotted in origin software and are exponential and sigmoidal, respectively.

The pressure profiles can be seen in Figure 1a‐b. Overall, the synthetic filters experienced lower pressure. In the primary filters this is due to the additional two layers present in the synthetic compared to the cellulose filters. In previous work,^23^ the secondary‐synthetic filters had a ~ 30% increase in max capacity (based on max. pressure of 2 bar) compared to the secondary‐cellulose filters. The capacity of secondary‐cellulose here (101 L/m^2^) is similar to values obtained in previous work, which was 117 L/m^2^ at similar cell culture viability of 66%, at a cell density lower by 20%. In this study, the secondary‐synthetic filters did not reach max capacity. The lower pressure in the secondary‐synthetic filter clearly shows that the additional layers in primary‐synthetic provide significant solids protection for the subsequent secondary filters.

### 3.2 Filtrate clarity (OD600) and DNA breakthrough

OD600 was used as a measure of filtrate clarity. Based on the pressure of the primary‐synthetic filters and the additional layers, an increased solids handling capacity and filtrate clarity was expected. However, the OD600 data suggest otherwise. A measurable difference is seen in OD600 absorbance in the primary filter breakthrough curves, which is a concern as it affects the secondary filter, as seen in Figure 1c‐d. There is an immediate breakthrough from the primary‐synthetic filters, which is higher than the primary‐cellulose filter. There is an overlap of the cellulose and synthetic (high) data around 50 L/ m^2^. Up to this point, OD of cellulose is increasing faster, where as OD of synthetic (high) has been more stable, but has started to increase after ~50 L/m^2^ reaching a final value of 56% higher than it's starting point and the final cellulose OD. The higher loading in primary‐synthetic filter also leads to reduced filtrate quality in secondary‐synthetic filter. Both filter types have the same nominal pore rating, and differences in cell culture conditions are unlikely different enough to potentially cause this breakthrough. In previous work, higher OD600 absorbance was observed from the secondary‐synthetic filter but only at viabilities below 48%. The viability of the cell culture here is between 67–75%. This leads to the conclusion that while nominal pore size rating is the same, there are some difference in the filter composition leading to a more challenging filtrate. One key difference is the composition of the filler material which has been changed from diatomaceous earth to silica, which is likely to have different absorbent properties.

The properties of cellulose and DE are better documented,^25^, ^26^ however with limited information about the newer synthetic materials it is difficult to make any conclusions. Polymeric binders also play important roles in the charge characteristics of depth filters, however specific information is propriety. A recent study, investigated the properties of D0HC (primary‐cellulose) and X0HC (secondary‐cellulose) for their ability to remove product‐related impurities, specifically low and high‐molecular weight species.^27^ They showed that retention of impurities on the primary filter were minimal, due to the open structure and absence of DE. Retention on the secondary filter was due to a combination of hydrophobic, electrostatic and hydrogen bonding and also dependant on the properties of the target impurity.

Overall, HCP is not being significantly removed by primary filters; this agrees with previous work. There is almost immediate breakthrough in both primary and secondary at these high cell densities (data not shown). Other studies have shown that the synthetic‐secondary filter has improved HCP removal and attributed it to the charge effects. However they have been done using model proteins only.^28^ It is hypothesized that at high cell density the electrostatic binding sites are not accessible for HCP retention due to the complex feed material blocking them.

DNA breakthrough can be seen in Figure 1e‐f. In the primary filter, the breakthrough is immediate, and there is no difference based on filter type. The breakthrough in the secondary filters depends on the DNA concentration at the input, which agrees with data from previous work. The secondary‐cellulose and secondary‐synthetic have similar filter loading and final DNA concertation in the pool, indicating that the additional layers in primary‐synthetic do not help retain extra DNA in the primary filter. This is also shown by similar DNA concentration in the intermediate pool. This does not match the initial hypothesis, where more DNA was expected to be removed by the synthetic filter train due to extra layers in primary‐synthetic and the protection they offer for the subsequent secondary‐synthetic filters.

Overall there has been a decrease in filtrate quality by changing to the synthetic filter train, which has also been linked with precipitation in the Protein A feed. The OD600 has been indicative of differences between the three harvest options and it was determined that the filtrates are different enough to proceed with the Protein A chromatography lifetime study.

## 4 CONFOCAL IMAGES AND ANALYSIS OF DEPTH FILTERS

### 4.1 Primary depth filters

**FIGURE 2:**
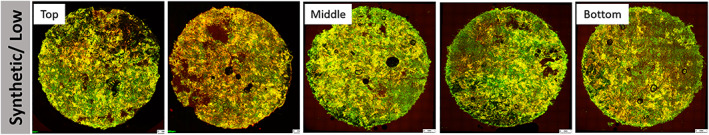
Example of confocal images where depth filters samples were stained with PicoGreen and Nile Red. This example corresponds to Layer 2 in the primary‐synthetic filter. The top, middle and bottom represent slices taken from the layer in the direction of flow and correspond to the integrated density in Figures 3 and 4. Each sample is 11 mm in diameter.

Each layer in the depth filter is approx. 4 mm thick hence it required sectioning before imaging under the microscope. Figure 2 shows examples of the images obtained, taken from top to bottom of each layer, in the direction of flow. Each section is approx. 60‐100 μm thick.

**FIGURE 3:**
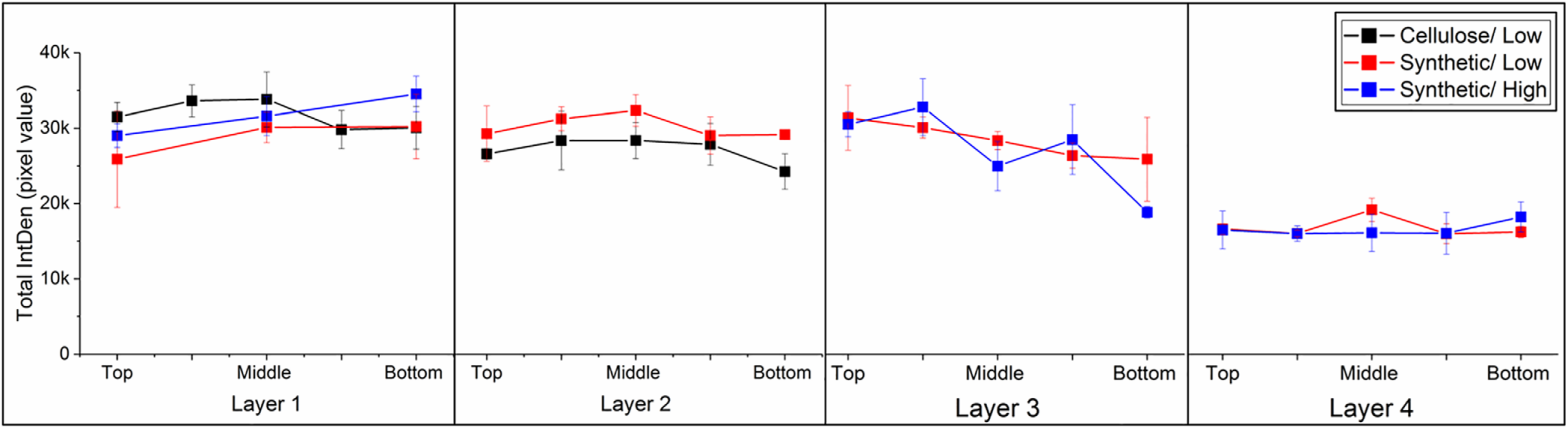
Total integrated density (sum of PicoGreen and Nile Red Integrated Density) across the depth of the primary depth filters. Error bars indicate 1SD of 3 measurements. The primary ‐ cellulose filter (black) is only composed of 2 layers, whereas the primary‐ synthetic (red and blue) have 4 layers. Low and High refers to filter loading of which details can be found in Table 1 and Figure 1. Data for Layer 2 Synthetic/High loading was not included as it was considered an inconclusive sample.

Confocal imaging and quantification were performed as per the method described here.^23^ Total Integrated Density (sum of PicoGreen and Nile Red intensity) was calculated for the primary depth filters as both dyes stained whole cells. Hence, data presented in Figure 3 describes total cells and cell debris distribution within the layers. This data aims to provide trends in foulant distribution rather than quantification of the foulant.

Quantitative data from the primary depth filters can be seen in Figure 3, where the x‐axis represents the direction of flow through the filters. For the 2‐layer primary‐cellulose filters, it can be observed that both layers are relatively full, and the foulant distribution is even across both layers. Data of layer 2, synthetic/high loading was not included as it was considered to be non‐representative and an artifact of sampling. Overall, in the 4‐layer primary‐synthetic filters, the foulant is distributed slightly differently. The first two layer are designed to capture cells and the large debris seen from the Total IntDen measurements showing the layers to be full. In layer 3 and 4, the intensity starts to drop through the depth of the filter. Lower intensity measurements in layer 4 are also likely to be due to finer particles.

### 4.2 Secondary depth filters

**FIGURE 4:**
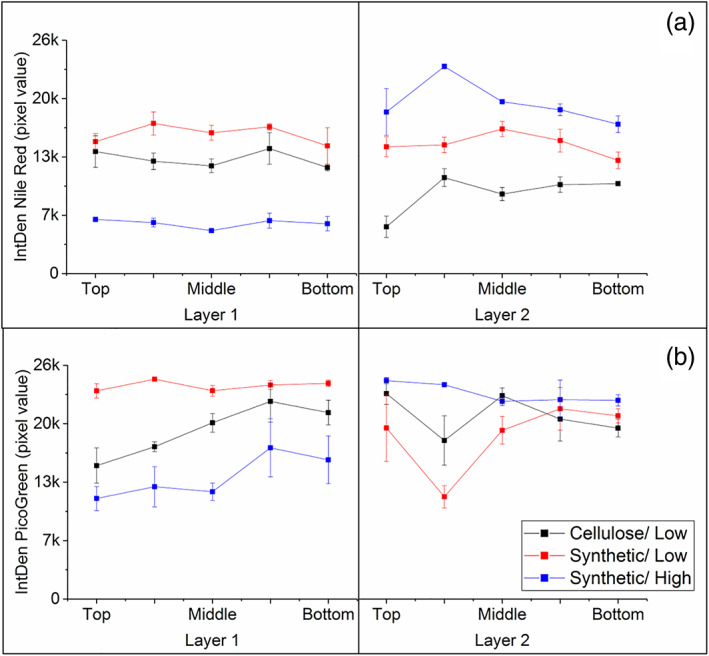
Integrated density of Nile Red (a) and PicoGreen (b) across the depth of the secondary depth filters. Error bars indicate 1SD of 3 measurements. Low and High refers to filter loading and details can be found in Table 1 and Figure 1.

The integrated density in the secondary filters also differs based on the filter type and loading, particularly the Nile Red, which describes cell debris, aggregates, etc. Nile Red distribution (Figure 4a) is similar in layer 1 of the secondary‐cellulose and the secondary‐synthetic at low loading. However at higher loading, there is a shift of foulant from layer 1 to layer 2 in the secondary‐synthetic filter.

Figure 4b shows that DNA retained across the secondary‐cellulose filter is constant across both layers. The drop in the second sample in layer 2 is likely to be an outlier. In layer 1 of the secondary‐synthetic filters, there is a gradual increase in DNA retained across the depth of the filters. This may relate to the distribution of DNA size and retention in the depth filter as described in literature.^29^ The trend of foulant movement further into the filter with increased loading is also seen with the DNA in the secondary‐synthetic filters, as with the Nile Red data.

There is not a clear relationship between the IntDen and DNA retained (calculated based on the data in Table 2). It is important to note, the imaging method aims to provide trends in the data rather than rather than quantification of the impurities retained.

**TABLE 2:** Impurities levels at different harvest stages, reported per IgG concentration. [HCP] = [Total Protein] – [IgG]

	Cell culture			Intermediate pool			Final filtrate		
Filter train	HCP/IgG (mg/mg)	DNA/IgG (μg/mg)	OD600 (AU)	HCP/IgG (mg/mg)	DNA/IgG (μg/mg)	OD600 (AU)	HCP/IgG (mg/mg)	DNA/IgG (μg/mg)	OD600 (AU)
**A**	4.5 ± 0.5	12.3 ± 0.5	0.33	4.2 ± 0.0	7.5 ± 0.6	0.08	2.9 ± 0.1	2.4 ± 0.1	0.05
**B**	3.0 ± 0.0	14.6 ± 0.5	0.29	3.0 ± 1.2	7.5 ± 0.4	0.09	3.0 ± 0.1	1.9 ± 0.1	0.08
**C**	1.9 ± 0.0	11.3 ± 0.8	0.31	1.7 ± 0.6	8.0 ± 0.5	0.1	1.4 ± 0.1	3.9 ± 0.1	0.08

In summary, the pressure profiles were as expected, and no significant HCP removal was observed. However, DNA removal was different than the hypothesis where secondary‐cellulose and secondary‐synthetic at similar loading removed a similar mass of DNA, even though the synthetic filtration train had a higher actual surface area. Additionally, the lower filtrate quality seen from the synthetic filters was not as predicted by the hypothesis. Therefore, it is believed differences in structure are likely to cause the higher solids breakthrough in the synthetic filters, which is supported by the confocal data where we see different foulant distributions based on filter type.

### 4.3 Protein A resin lifetime

Following the harvest step, the Final Filtrate was aliquoted and frozen for Protein A resin lifetime study. Experiments were conducted with 1 ml HiTrap column prepacked with MabSelect Sure LX, and 100 cycles were performed for each condition. Based on the results of the depth filtration, conditions A and B were expected to give similar results, while condition C was the worst case in this scenario. Attributes measured were eluate peak broadening, DBC and impurity levels in the eluate (HCP and DNA). The residence time was 0.75 min (typically it is 3‐6 min), it was a necessity in the experimental design due to time limitations of the cycling studies. It is expected that fouling would have been more significant with longer residence time, however the results are still comparable as all three columns were operated in the same way. Further, after 100 cycles, the resin was imaged with two dyes, Proteostat and PicoGreen to visualize fouling.^24^, ^30^

While thawing the aliquots, significant precipitation levels were observed in Feed B and C but not in Feed A. These precipitates are linked with the poor filtrate quality seen during harvest using synthetic depth filters. OD600 measurements of the Feeds were 0.023, 0.104, and 0.115 for Feeds A‐C, respectively. The composition of these precipitates is unknown, likely a higher level of process‐related impurity coming through and/or a leachable from the synthetic filters. They were visually observed to be white and large enough to block AKTA tubing. Hence Feed B and C were centrifuged before syringe filtration and loading onto the column. After centrifugation, the OD600 was 0.015 and 0.020 for Feed B and C, respectively.

No significant trends were observed from the Protein A performance in terms of eluate peak broadening, DBC and impurity levels in the eluate. This is likely due to CIP after each cycle and the precipitation leading to removal of some components from the loading material.

### 4.4 Resin imaging and fouling

**FIGURE 5:**
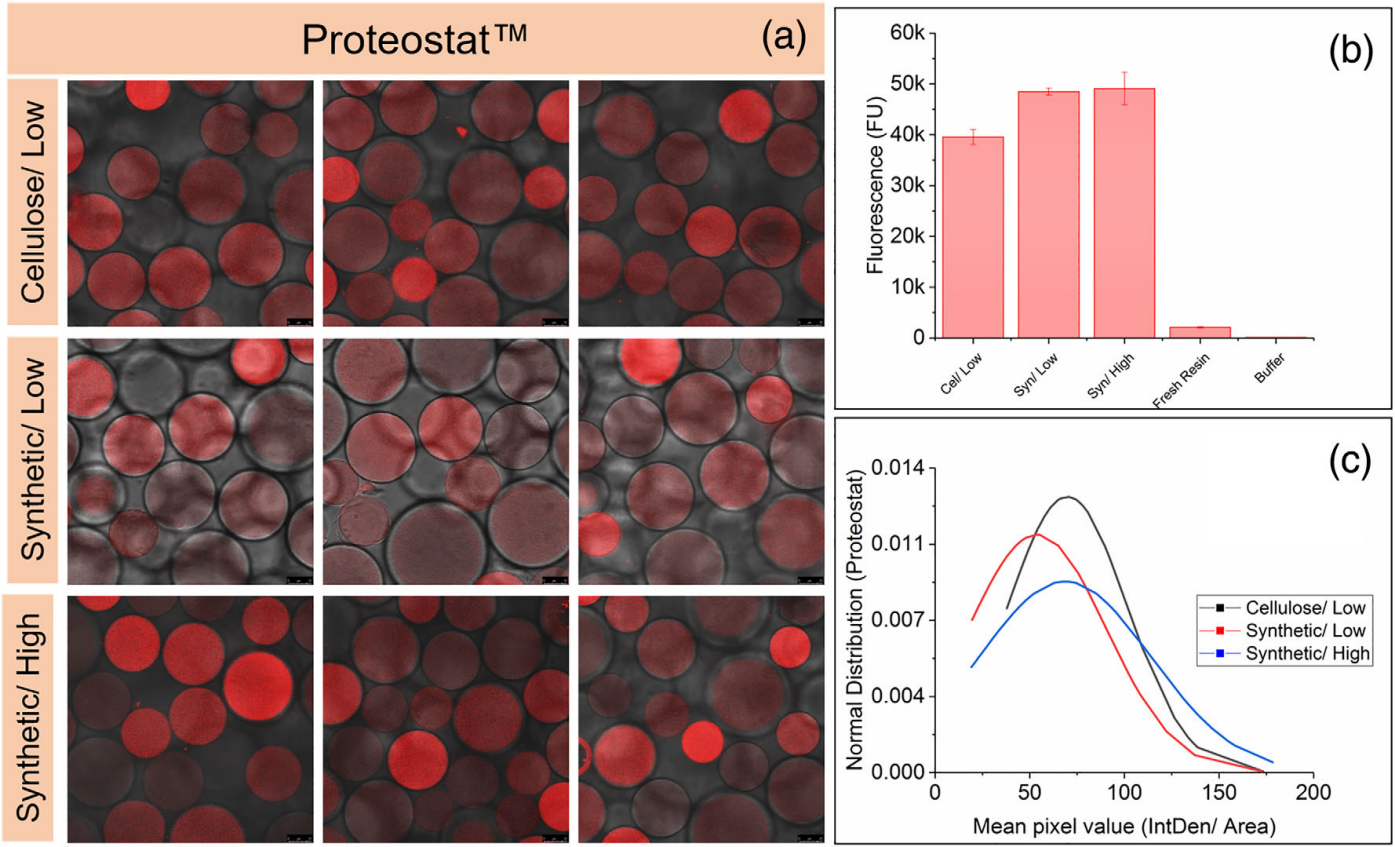
(a) Overlaid CLSM and bright‐filed images of fouled resin after 100 cycles stained with Proteostat™. Three images for each column represent 3 different samples. (b) Fluorescence measured by plate‐assay using Proteostat™, where error bars represents 1SD of 3 assay repeats. (c) The normal distribution of the mean pixel value based on CLSM images.

**FIGURE 6:**
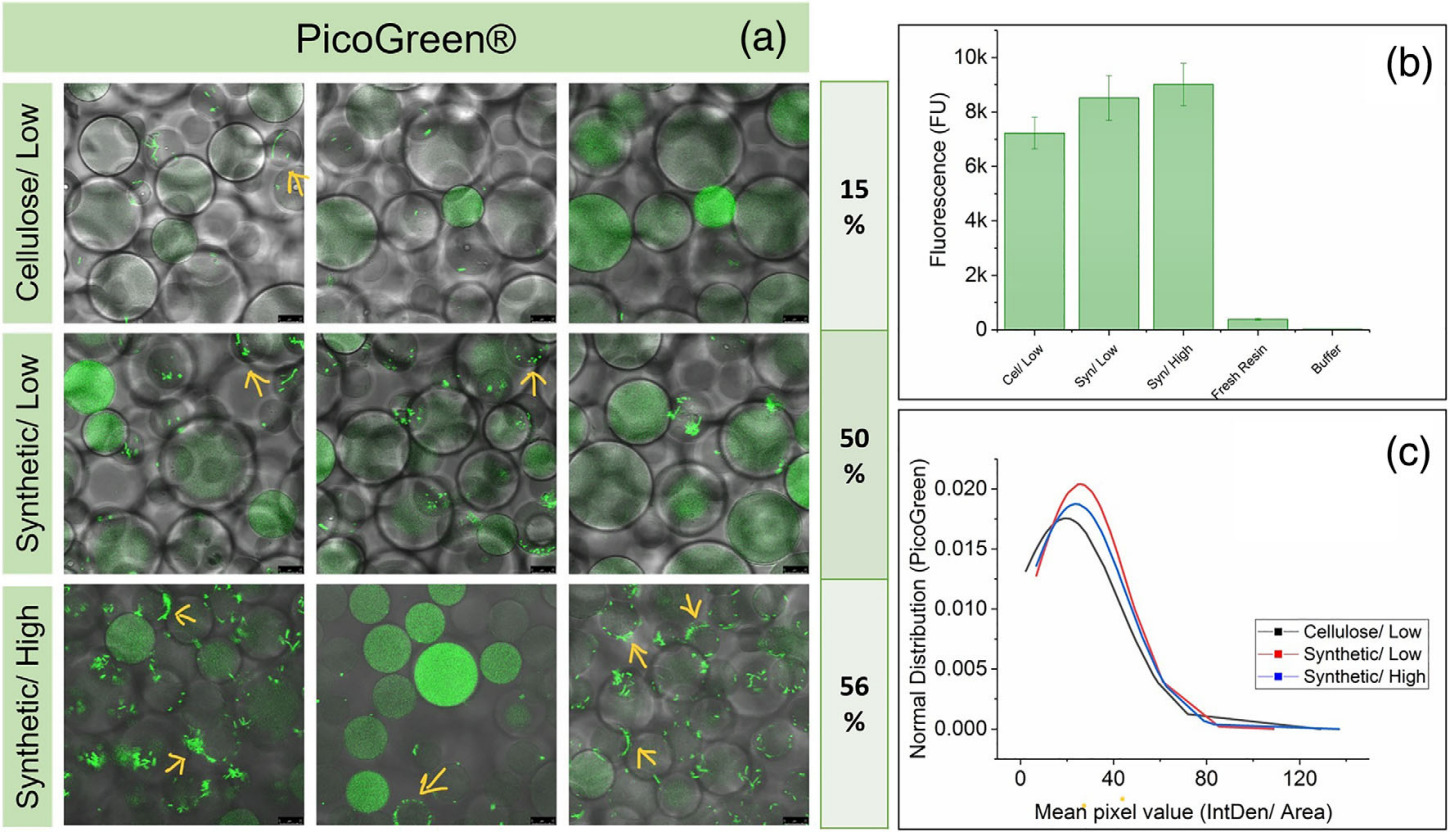
(a) Overlaid CLSM and bright‐filed images of fouled resin after 100 cycles stained with PicoGreen®. Three images for each column represent 3 different samples. The percentage values indicate the percentage of beads in the sample which have DNA build‐up on the surface of the bead. (b) Fluorescence measured by plate‐assay using PicoGreen®, where error bars represents 1SD of 3 assay repeats. Yellow arrows highlight examples of DNA build up on the resin. (c) The normal distribution of the mean pixel value based on CLSM images.

After the 100th cycle, the resin was removed from the column and resuspended. Three samples of the resin were stained with Proteostat and PicoGreen individually. PicoGreen becomes fluorescent when it intercalates with dsDNA. The Proteostat dye fluoresces upon binding to protein aggregates.^31^, ^32^, ^33^ Figures 5 and 6 show the confocal and brightfield overlapped images. The Proteostat method, developed previously,^24^ was adapted for PicoGreen. Negative control with clean resin showed no binding of the dye as observed from the confocal images for both Proteostat and PicoGreen dyes. Plate fluorescence measurements of the three resins are also shown in Figures 5b and 6b for Proteostat and PicoGreen, respectively. Based on the plate fluorescence measurement, columns B and C (synthetic harvest train) experinced higher fouling then column A (cellulose harvest train).

As seen in Figures 5a and 6a, some beads had a higher flourescence intensity than others and differed in size. Therefore it was decided to measure the Mean Integrated Density, the sum of the total pixel value per area selected. Normal distribution was plotted for both dyes. Higher intensity beads are believed to be from the top of the column, having experienced the most fouling, which agrees with the literature.^24^, ^34^ The normal distribution shows a broadening of the curve and a shift to the right for column C, where column A is considered the baseline, indicating that more of the beads in the sample have a higher fluorescence, suggesting a higher level of aggregates present on the resin.

The distribution of bound DNA is different from the protein aggregates, which seen visually in Figure 6a. Based on the Normal Distribution and the plate fluorescence data, the amount of fouling is not significantly different between the cellulose and synthetic filter train. However the main difference is the nature of the fouling, with the formation of DNA rings outside the beads. It is hypothesized this is genomic DNA which has build‐up around the beads. This can potentially lead to mass transfer difficulties and hence reduced DBC.

PicoGreen –plasmind DNA had been used to show the shrinking core model of binding on Q Sepharose FF resin.^35^ A major concern in literature is chromatin binding onto Protein A chromatography resin. It has been shown that chromatin forms hetteroaggregates with histones, leading to resin fouling, accumulating on particle surfaces and obstructing IgG accesss to the resin pores.^5^, ^6^, ^8^, ^10^, ^36^, ^37^, ^38^, ^39^ However most of these studies have been done with cell culture at 20–50% viability, not representing a manufacturing setting.

## 5 CONCLUSION

The choice of depth filters during the harvest of CHO cell culture can affect the downstream unit operation, such as Protein A chromatography. A change from cellulose + DE to fully synthetic materials was expected to have a positive effect on the process, with claims of improved HCP removal^19^ due to the charged nature in the secondary‐synthetic filter and the two additional layers in the primary‐synthetic filter. The larger actual surface area in the primary‐synthetic led to reduced pressure in both primary and secondary depth filter stages, indicating that it provides a protective nature to the secondary filter downstream. However, this did not relate to any benefits in terms of impurity removal, either in terms of DNA or HCP. Unexpectedly, the synthetic filter trains were associated with decreased filtrate quality and significant precipitation in the Protein A feed. In addition, confocal imaging showed foulant was pushed down through the filter with higher loading. Imaging also showed the build‐up of DNA deposits on the resin was more significant when synthetic depth filters were used at harvest.

The results of this study have highlighted the need for further knowledge and a deeper understanding of depth filtration processes. This is particularly important if the industry is looking to move away from traditional cellulose and DE materials, whose suitability has been historically established through empirical methods. The application of breakthrough curves and imagining techniques can provide new information regarding the fouling behavior of depth filters and resin. As shown in this study, the formation of the DNA rings on the resin was associated with using synthetic filters in the harvesting step, with the potential to affect mass transfer hence DBC, resin lifetime, and costs.

## AUTHOR CONTRIBUTIONS

**Maria Parau:** Conceptualization (equal); data curation (lead); formal analysis (lead); investigation (lead); methodology (lead); project administration (lead); writing – original draft (lead); writing – review and editing (equal). **James Robert Pullen:** Conceptualization (equal); funding acquisition (equal); resources (equal); supervision (equal); writing – review and editing (equal). **Daniel Gilbert Bracewell:** Conceptualization (equal); funding acquisition (equal); methodology (equal); project administration (equal); resources (equal); supervision (equal); writing – review and editing (equal).

## CONFLICT OF INTEREST

Authors have no conflict of interest.

### PEER REVIEW

The peer review history for this article is available at https://publons.com/publon/10.1002/btpr.3329.

## Data Availability

Data available on request from the authors
